# Profiles and predictors of mental health of university students in Hong Kong under the COVID-19 pandemic

**DOI:** 10.3389/fpsyg.2023.1211229

**Published:** 2023-07-18

**Authors:** Daniel T. L. Shek, Wenyu Chai, Xiang Li, Diya Dou

**Affiliations:** Department of Applied Social Sciences, The Hong Kong Polytechnic University, Kowloon, Hong Kong SAR, China

**Keywords:** mental health, university students, risk and protective factors, COVID-19 pandemic, Hong Kong

## Abstract

This study investigated the mental health problems of university students in Hong Kong and related sociodemographic and psychosocial predictors under the pandemic. A total of 978 undergraduate students (mean age = 20.69 ± 1.61) completed an online questionnaire measuring sociodemographic factors, psychological morbidity, positive well-being, COVID-19 related stress and self-efficacy, and positive psychosocial attributes. Psychosocial risk factors included psychological morbidity, COVID-19 related stress, and difficulties encountered under the pandemic, whereas protective factors comprised pandemic related self-efficacy, positive psychological attributes, positive environmental factors, need satisfaction and positive perception toward service. Results showed that psychological morbidity in the participants was widespread, and it was related to sociodemographic factors, particularly family financial difficulties. While pandemic related stress positively predicted psychological morbidity and negatively predicted well-being indicators, COVID-19 self-efficacy showed an opposite effect. Besides, positive psychological attributes (resilience, emotional competence, and positive beliefs related to adversity) and environmental factors (healthy family functioning, peer support, and supportive community atmosphere) negatively predicted psychological morbidity and positively predicted well-being. Furthermore, need satisfaction and positive perception toward service were negatively associated with psychological morbidity and positively associated with well-being, while perceived difficulties showed an opposite effect.

## 1. Introduction

### 1.1. Mental health in university students under the pandemic

Since its first outbreak in 2019, the COVID-19 pandemic has dramatically changed our lives, which has negative impacts on people’s physical and mental health (Asmundson et al., 2020; Shek, 2021; Rehman et al., 2023; Shek et al., 2023c). In Hong Kong, the COVID-19 pandemic reached a turning point during the fifth wave from 31 December 2021 to 31 May 2022 (Wong et al., 2023). Before the fifth wave, Hong Kong had almost no local cases of COVID-19 for around 3 months due to the very stringent anti-epidemic measures (Cheung et al., 2022). However, the reported confirmed cases reached 1,200,068 during the fifth wave, which was 95 times higher than the first four preceding waves (Wong et al., 2023). Also, the rapid increase in cases led to more stringent measures used to control the spread of the virus. The adverse environment undoubtedly exacerbated the stress, anxiety, and fatigue of Hong Kong citizens (Lau et al., 2022), which led to increased mental health problems (Lo et al., 2023).

With specific reference to university students, they may suffer more from the pandemic compared with other groups (Dragioti et al., 2022) because of the sudden changes in learning requirements and reduction in social activities. A study based on 1,000 Greece undergraduate students showed elevated depression, anxiety, and suicidal ideation during the lockdown (Kaparounaki et al., 2020). Another study in the United Kingdom (*N* = 1,173) also revealed high levels of depression and anxiety under the pandemic (Chen and Lucock, 2022). In addition, Savage et al. (2020) showed that students’ mental well-being deteriorated during the lockdown period. Shek et al. (in press) have also summarized the findings on the deteriorating student mental health profiles in the early days of the pandemic. In the present study, we attempted to examine the mental health and related sociodemographic as well as psychosocial predictors in Chinese university students in Hong Kong under the pandemic. In this study, mental health was conceived in terms of psychological morbidity and positive well-being. While psychological morbidity was indexed by depression, anxiety, stress, post-traumatic stress disorder (PTSD), Internet addiction, suicidal behavior, and hopelessness, positive well-being measures included life satisfaction and flourishing in this study.

### 1.2. Theoretical framework guiding the study

The ecological systems theory of Bronfenbrenner provides a comprehensive framework for understanding the interactions between an individual and his/her respective environments, as well as the effects of these interactions on human well-being and mental health (Bronfenbrenner, 1979). This theory proposes that multiple interconnected systems shape human development involving the individuals and their environment (Bronfenbrenner, 1979). Guided by this theory, the stresses and difficulties faced by university students under the pandemic constitute risk factors for the psychological well-being of the students. This study also incorporates a strengths-based perspective, which emphasizes the significance of identifying and cultivating individual and environmental strengths that play a vital role in protecting human health in the face of adversity such as the pandemic. For example, Benson (2007) proposed 40 developmental assets that identify positive experiences, attributes, and qualities that promote positive youth development. The 40 developmental assets are divided into two major categories: external assets and internal assets. External assets highlight the positive experiences and support that young people receive from their environment, such as family, peers, and universities. Internal assets are the qualities and competencies that young people possess within themselves, such as positive values and social competencies. There are also views suggesting the importance of positive psychological attributes in promoting well-being under the pandemic (Shek, 2020; Shek et al., 2021). By integrating these two perspectives, the present study aimed to investigate the prevalence of mental health problems, as well as the associated sociodemographic predictors and psychosocial protective and risk factors among Hong Kong university students under the COVID-19.

### 1.3. Sociodemographic predictors of mental health

At the individual level, research showed that female students had a higher level of mental health symptoms than did male students under the pandemic (Patsali et al., 2020; Prowse et al., 2021). Regarding age, while younger students reported higher mental health problems (Sequeira et al., 2022; Kondo et al., 2023), some studies showed that older students exhibited relatively poorer mental health (Chi et al., 2020). Husky et al. (2020) also showed that students living alone were more vulnerable than students in other living status. In addition, students with family members or relatives infected with COVID-19 showed a higher level of mental health problems (AlHadi and Alhuwaydi, 2021). Furthermore, studies showed that financial problems positively predicted mental health problems (Armiya’u et al., 2022; Nadareishvili et al., 2022; Galanza et al., 2023). Based on the literature, we examined several sociodemographic correlates in this study: age, gender, living status, student identity (local or international), family and personal financial difficulties, and family and personal infection experience.

### 1.4. COVID-related stress and self-efficacy as predictors of mental health

The outbreak of COVID-19 has created stressors such as fear of contracting the disease and concerns about the implementation of strict quarantine and isolation policies. The literature suggests that COVID-related stress and threats are risk factors for mental health in tertiary students and young adults (Moroń et al., 2021; Sun et al., 2021; Faisal et al., 2022; Green and Yıldırım, 2022). At the same time, a higher level of perceived self-competence in addressing pandemic-related issues (i.e., self-efficacy related to COVID-19) protects mental health and promotes well-being in university students (Sun et al., 2021; Lai et al., 2022). Particularly, COVID-related self-efficacy, such as the belief in one’s ability to handle situations and adapt to challenges related to the pandemic, can have a significant effect on the psychological well-being of students (Brailovskaia and Margraf, 2022).

### 1.5. Positive psychological attributes as predictors of mental health

Researchers have found that positive psychological attributes, such as resilience, emotional competence, and beliefs related to adversity negatively predicted psychological morbidity, including depressive symptoms and anxiety under the pandemic (Chi et al., 2020; Krifa et al., 2022). Existing research has demonstrated the function of resilience as a stress buffer during psychological crises (Chi et al., 2020; Chan A. C. et al., 2021). Additionally, individuals with a strong belief about their ability to overcome adversity possess confidence in their ability to confront and manage challenges, maintain a positive outlook, and grow from their experiences (Dweck and Yeager, 2019). Furthermore, emotional competence is identified as a positive strength that protects the mental health of university students under the pandemic (Favieri et al., 2021; Hu et al., 2023). For example, Wang et al. (2021) found that under the pandemic, students with better emotion regulation skills experienced lower anxiety and depression.

### 1.6. Environmental predictors of mental health

At the environmental level, research also highlighted the importance of external assets at the microsystem level to protect university students’ development (Suresh et al., 2021; Zeng et al., 2021). Healthy family functioning and peer and community support predicted lower depressive symptoms and anxiety (Sun et al., 2020; Nadareishvili et al., 2022). For instance, Nadareishvili et al. (2022) discovered that fewer family conflicts were negatively associated with anxiety and depression in American university students under the pandemic. Ye et al. (2020) emphasized the importance of social support, including community support, in reducing stress and boosting resilience among college students during the pandemic. Elmer et al. (2020) also reported that students who maintained social connections and received community support had better mental health outcomes than those who were more socially isolated under the pandemic.

### 1.7. Need satisfaction, perceived difficulties and service utilization

Disruptions in academic study and social isolation have increased the difficulties for university students during the pandemic and may further negatively affect their mental health (Salimi et al., 2023). In addition, students’ need satisfaction and satisfaction with university services were also positively related to their well-being under the pandemic (Lai et al., 2022; Shek et al., 2022b). According to Deci and Vansteenkiste (2004), one best understanding of how the interaction between individual and his/her environment shapes human development is to examine “the degree to which the environment thwarts versus satisfied people’s basic psychological needs” (p. 24). They argued that satisfaction of basic psychological needs through person-environment interaction contributes to positive well-being and prevents mental health illness. Therefore, need satisfaction under the pandemic can be considered as an important protective factor for mental health of university students. As such, tailor-made university services and resources are critical external assets for students to address their difficulties and satisfy their basic psychological needs under the pandemic (Lai et al., 2022). Previous studies have found that perceived usefulness as well as evaluation of university services were negatively related to their mental health in the pandemic period (Lai et al., 2022; Shek et al., 2022b).

### 1.8. Research questions of the study

Based on the above literature review, the present study investigated the mental health profiles and related sociodemographic as well as psychosocial predictors in Hong Kong university students in the COVID-19 period. With reference to the guiding conceptual framework, we asked several specific research questions and proposed related hypotheses as below.

Research Question 1: What is the mental health condition in Hong Kong university students under the COVID-19 pandemic? We expected that the prevalence of mental health problems would be on par with those reported in the local (Shek et al., 2021, 2022a) and international context (Kaparounaki et al., 2020; Chen and Lucock, 2022).

Research Question 2: Under the pandemic, what is the relationship between sociodemographic factors and mental health of university students? Particularly, as financial difficulty is a prevailing problem under the pandemic (Nadareishvili et al., 2022; Galanza et al., 2023), we proposed that financial difficulties would be positively linked to psychological morbidity (Hypothesis 1a) and negatively linked to positive well-being in university students (Hypothesis 1b).

Research Question 3: With specific focus on COVID-19, what are the relationships between perceived COVID-19 stress and self-efficacy as well as mental health of university students? In line with the literature (Sun et al., 2021; Yıldırım and Arslan, 2021; Faisal et al., 2022), we hypothesized that (a) perceived COVID-19 stress would positively predict psychological morbidity (Hypothesis 2a), (b) perceived COVID-19 stress would negatively predict well-being (Hypothesis 2b), (c) COVID-19 self-efficacy would negatively predict psychological morbidity (Hypothesis 2c), and (d) COVID-19 self-efficacy would positively predict well-being (Hypothesis 2d).

Research Question 4: What are the relationships between positive psychological attributes (resilience, emotional competence, beliefs about adversity) and student mental health under the pandemic? Based on the literature (Chi et al., 2020; Krifa et al., 2022; Shek et al., 2022b), we expected that positive psychological attributes would negatively predict psychological morbidity (Hypothesis 3a), and positively predict well-being (Hypothesis 3b).

Research Question 5: What are the associations between environmental factors (including family functioning, peer support, and community atmosphere) and student mental health under the pandemic? Based on the existing literature (Sun et al., 2020; Nadareishvili et al., 2022), we expected that supportive environmental factors would negatively predict psychological morbidity (Hypothesis 4a) and positively predict well-being (Hypothesis 4b).

Research Question 6: What are the associations between need satisfaction, perceived difficulties, and perceived usefulness of university services and student mental health under the pandemic? Referring to the existing literature (Kecojevic et al., 2020; Lai et al., 2022; Shek et al., 2022b), we hypothesized that need satisfaction and perceived usefulness of university services would be negatively associated with psychological morbidity while perceived difficulties would be positively associated with mental health problems (Hypothesis 5a), and need satisfaction and perceived usefulness of university services would be positively associated with well-being while perceived difficulties would be negatively associated with well-being (Hypothesis 5b).

**Figure 1:**
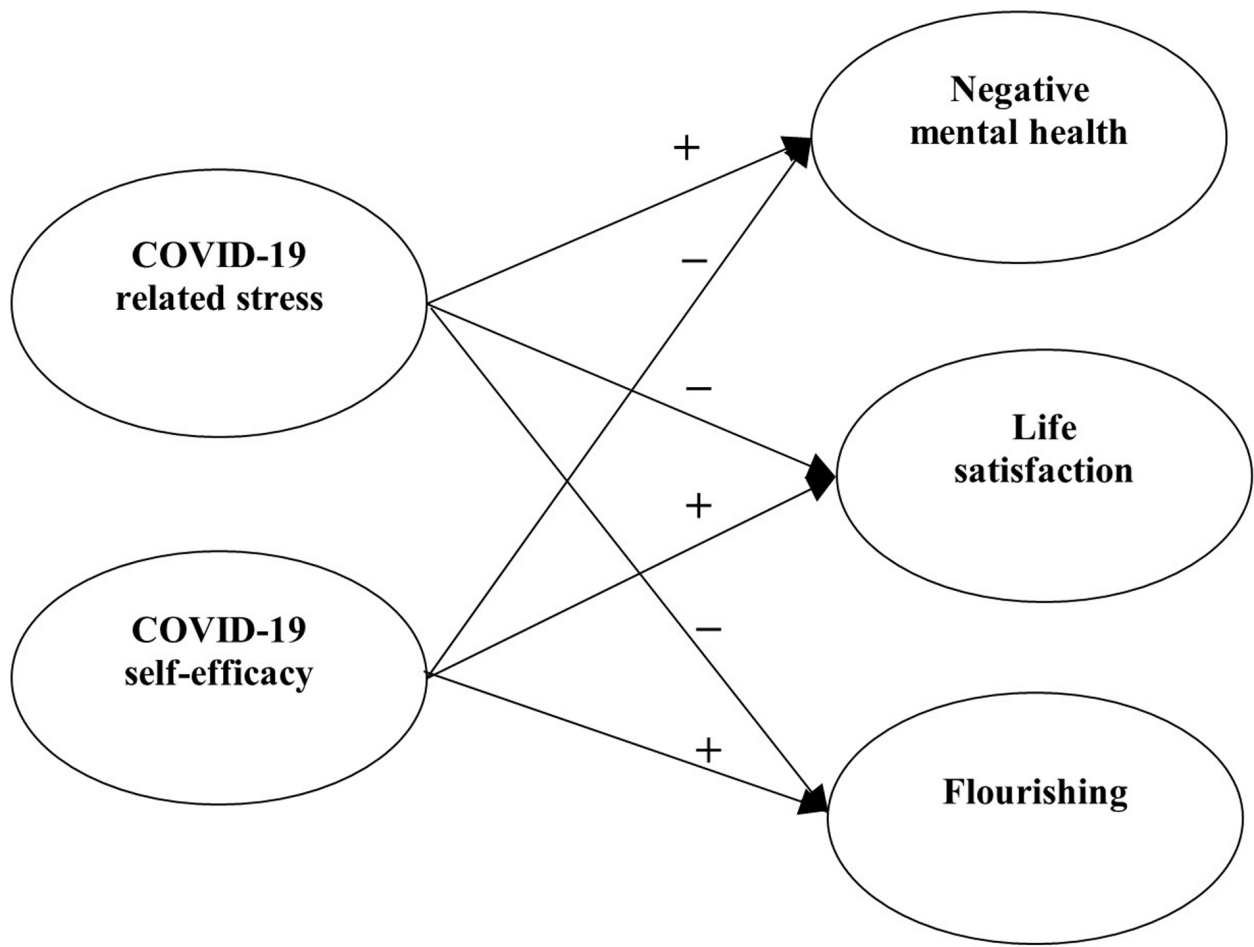
Conceptual model 1: predictive effects of COVID-19 related stress and self-efficacy on mental health.

**Figure 2:**
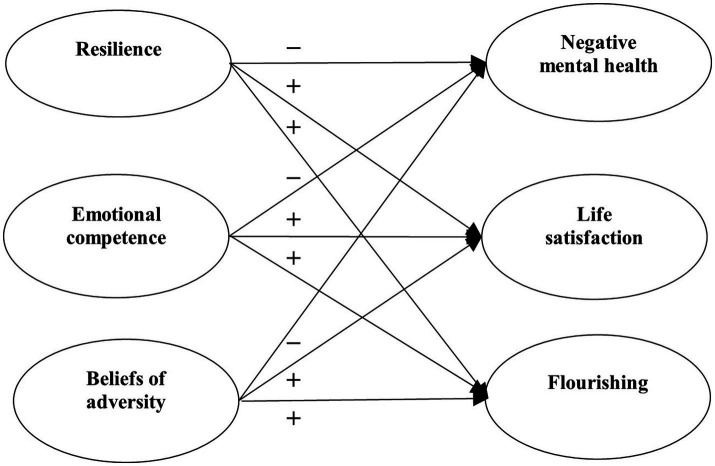
Conceptual model 2: predictive effects of positive psychological attributes on mental health.

**Figure 3:**
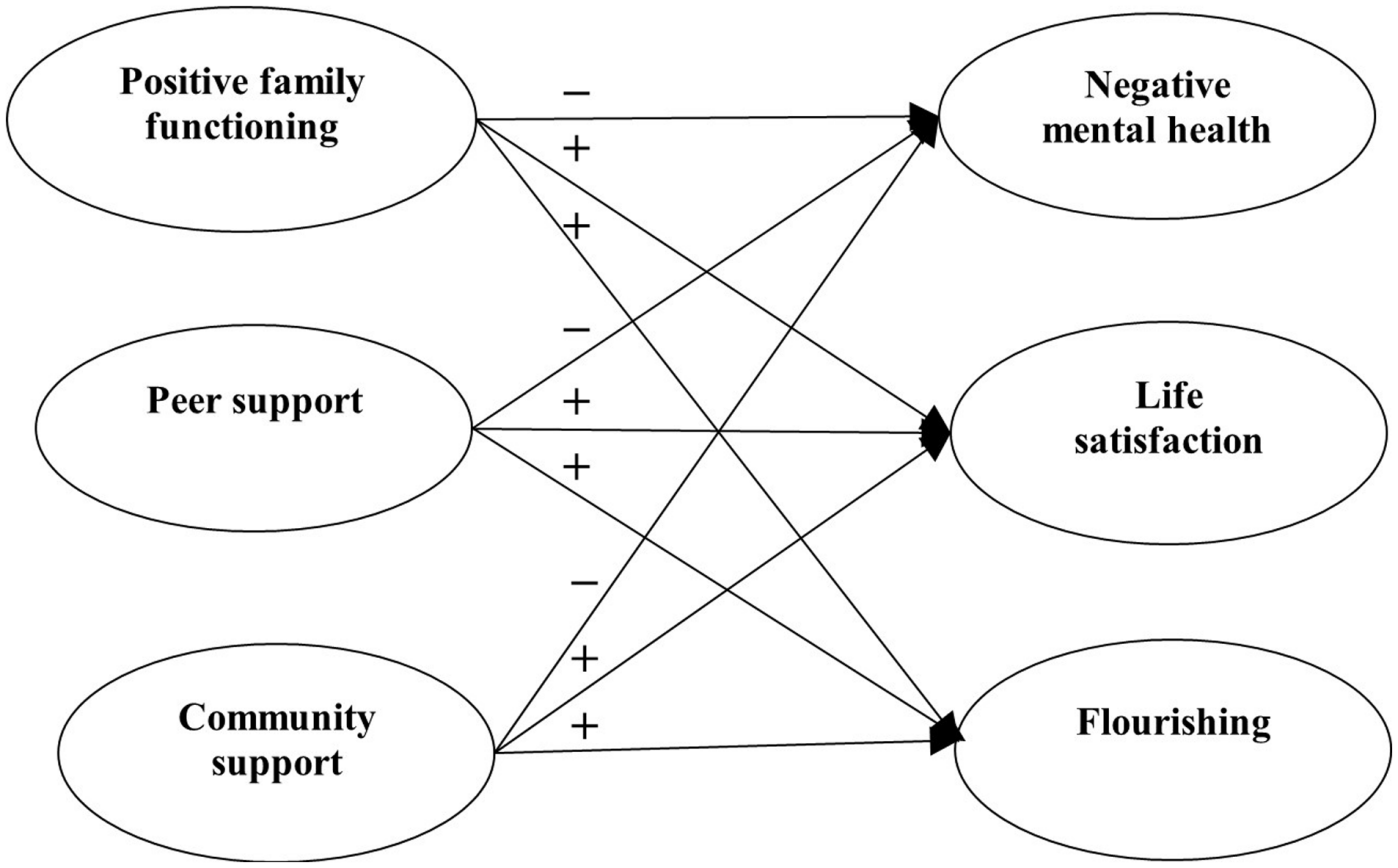
Conceptual model 3: predictive effects of environmental factors on mental health.

**Figure 4:**
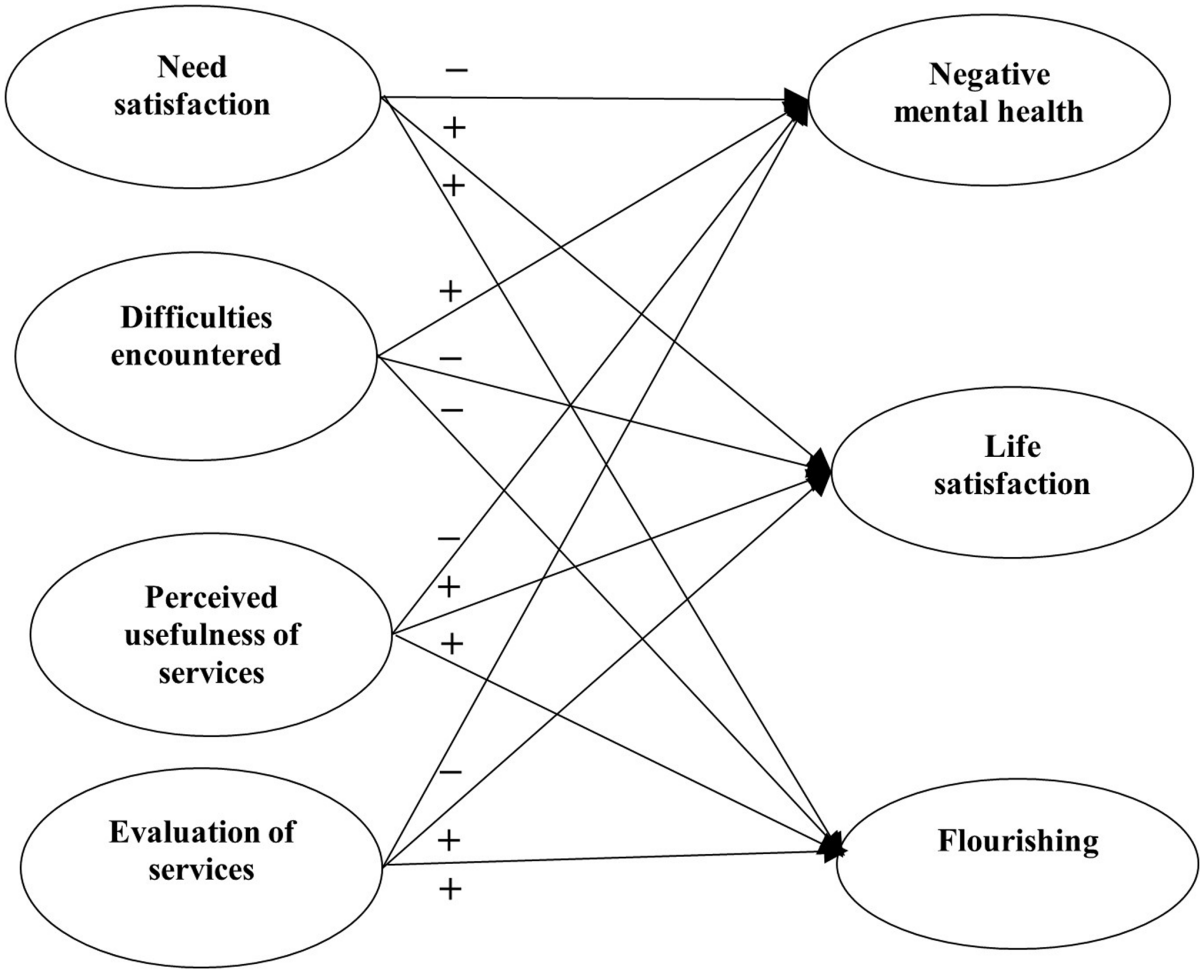
Conceptual model 4: predictive effects of need satisfaction, difficulties encountered, service usefulness, and service evaluation on mental health.

With reference to the above research questions, we proposed four separate models to be tested. For Model 1, the predictive effects of factors related to COVID-19 (i.e., COVID-19 related stress and self-efficacy) on mental health of university students were examined (Figure 1). For Model 2, the predictive role of positive psychological attributes (i.e., resilience, emotional competence, and beliefs about adversity) in university students’ mental health was investigated (Figure 2). In Model 3, the predictive role of environmental attributes (i.e., family functioning, peer, and community support) in mental health of university students was tested (Figure 3). In Model 4, the role of need satisfaction, difficulties encountered, perceived service usefulness, and service evaluation in the mental health of university students was explored (Figure 4). In each model, the mental health outcomes included three constructs: psychological morbidity, life satisfaction, and flourishing. As psychological morbidity refers to a pathological state in mental and behavioral aspects, it was indexed by a latent factor including depression, anxiety, stress, PTSD, Internet addiction, suicidal behavior, and hopelessness.

## 2. Materials and methods

### 2.1. Participants and procedure

In the summer of 2022 when the fifth wave of COVID-19 (Omicron strain) occurred in Hong Kong, we invited undergraduate students from a public university in Hong Kong to participate in an online survey. Due to the difficulties in conducting random sampling in the pandemic period, quota sampling was used to recruit the participants with faculty and year of study as two stratifying factors. As one type of non-probability sampling, quota sampling selects a sample from a population consisting of subgroups and selects participants from each subgroup (i.e., stratifying factor) based on a convenient approach (Rukmana, 2014; Futri et al., 2022). This sampling strategy was widely adopted in different studies conducted under the pandemic (McBride et al., 2021; Futri et al., 2022). A total of 978 undergraduate students (mean age = 20.69 ± 1.61; female = 62.9%) completed the online survey questionnaire using the Qualtrics XM platform. The questionnaire and the measures were in English because the participants were recruited from a university in Hong Kong in which English is the primary medium of instruction (e.g., teaching is primarily conducted in English and students are required to complete their assignments and examination mainly in English). Another reason is that most of the English measures used in this study had been validated in different cultures including Hong Kong. The sample characteristics are summarized in Table 1. The research was approved by the institutional ethics review board. All the study participants were notified of the research purpose and the confidentiality of collected data. They also gave their formal informed consent before participating in the survey.

**Table 1:** Socio-demographics characteristics of the participants in the final sample (N = 978).

Variable	*n*	*%*
Faculty		
Faculty of Engineering	164	16.8
Faculty of Construction and Environment	116	11.9
Faculty of Health and Social Sciences	325	33.2
Faculty of Applied Science and Textile	69	7.1
Faculty of Humanities	43	4.4
Faculty of Business	195	19.9
School of Design	30	3.1
School of Hotel and Tourism Management	36	3.7
Gender		
Male	336	34.4
Female	615	62.9
Prefer not to say	27	2.8
Year of study		
2nd 3rd 4th	418 322 238	42.7 32.9 24.3
Received CSSA Yes	45	4.6
No	905	92.5
Experienced financial difficulty (family)		
Yes	200	20.4
No	659	67.4
Experienced financial difficulty (personal)		
Yes	279	28.5
No	630	64.4
Students or their family members unemployed under the pandemic		
Yes	206	21.1
No	707	72.3
Student had been a confirmed case of COVID-19		
Yes	232	23.7
No	712	72.8
Family members had been a confirmed case of COVID-19		
Yes	358	36.6
No	584	59.7
Living status		
Live with family	870	89
Live with roommates	86	8.8
Live alone	22	2.2
Place of residence under the pandemic		
Hong Kong	925	94.6
Mainland China	38	3.9
Others	15	1.5
Place of origin (Local/International student)		
Local	917	93.8
International	61	6.2

CSSA, comprehensive social security assistance scheme.

### 2.2. Measures

#### 2.2.1. Psychological morbidity measures

##### 2.2.1.1. Depression Anxiety Stress Scale

Depression Anxiety Stress Scale (DASS-21) was adopted to assess three mental health problems: depression, anxiety, and stress, with each problem being assessed by seven items (Lovibond and Lovibond, 1995). Each participant reported his/her frequency of displaying the item-described symptom on a scale of four points (“0” = “*Not at all*” to “3” = “*Most of the time*”). By summing corresponding item scores, the total score of each problem was calculated. Previous studies supported the psychometric properties of the measure (Li X. et al., 2021; Cao et al., 2023). The information on the internal consistency (Cronbach’s alpha values) and the related statistics were reported in Table 2.

**Table 2:** Reliability, mean, and standard deviation of measures of different variables.

Measure	Cronbach’ *α*	Inter-item correlation	*M*	*SD*
Negative mental health				
Depression Anxiety Stress Scale (DASS-21)	0.95	0.45	1.62	1.07
Trauma Screening Questionnaire (TSQ)	0.82	0.31	3.22	2.84
Center for Epidemiologic Studies Depression Scale Revised (CESD-R)	0.96	0.55	16.04	14.12
Young’s 10-item Internet Addiction Test (IAT-10)	0.81	0.30	3.59	2.82
Suicidal Behavior Scale (SBS)	0.65	0.46	0.20	0.56
Chinese Hopelessness Scale (CHOPE)	0.86	0.55	3.30	0.91
Positive wellbeing				
The Satisfaction with Life Scale (SWLS)	0.88	0.60	3.54	0.98
The Flourishing Scale (FS)	0.93	0.63	4.62	1.09
COVID-19 related factors				
The Perceived COVID-19 Stress Scale				
Danger and contamination subscale	0.92	0.70	1.61	1.00
Socio-economic consequences subscale	0.90	0.65	1.00	0.87
Checking behavior subscale	0.84	0.51	1.63	0.79
Self-efficacy related to COVID	0.92	0.52	2.57	0.59
Positive psychological attributes				
Chinese Cultural Beliefs about Adversity Scale (CBAS)	0.76	0.27	3.97	0.68
Chinese Positive Youth Development Scale (CPYDS)				
Resilience subscale	0.82	0.61	4.12	0.86
Emotional competence subscale	0.80	0.58	4.08	0.92
Positive environmental factors				
Chinese Family Assessment Instrument (C-FAI, family functioning)	0.80	0.32	3.33	0.63
Multidimensional Scale of Perceived Social Support (MSPSS, peer support)	0.86	0.60	3.78	0.73
Community atmosphere measure	0.83	0.62	3.35	0.70
Need satisfaction, service evaluation, and difficulties				
Need Satisfaction	0.89	0.36	3.97	0.74
Perceived Usefulness of University Service	0.93	0.56	4.10	0.91
Evaluation of University Service	0.95	0.60	3.90	0.84
Difficulties Encountered	0.91	0.30	3.01	0.60

##### 2.2.1.2. The Center for Epidemiologic Studies Depression Scale Revised

Depressive symptoms were also assessed by Center For Epidemiologic Studies Depression Scale Revised (CESD-R), a 20-item measure assessing depression based on nine-cluster symptoms developed corresponding to the “American Psychiatric Association Diagnostic and Statistical Manual (DSM-V)” (Eaton et al., 2004). On each item, the participants rated the frequency of their displaying the described symptom from “0” = “*Not at all or less than 1 day*” to “4” = “*Nearly every day for 2 weeks*.” The sum of all item scores was calculated as the score of CESD-R. Previous studies supported the psychometric properties of the measure (Dou et al., 2021; Zhu et al., 2021).

##### 2.2.1.3. Young’s 10-Item Internet Addiction Test

Ten-Item Internet Addiction Test (IAT-10) (Young, 1998) was adopted to measure Internet addiction (IA), which consists of 10 items each describing one behavior/state related to problematic use of the Internet. Shek et al. (2008) validated the Chinese version of the scale. In this study, the participants reported whether they experienced the addictive symptoms described by each item over the past one-year time through a dichotomous rating (“1” = “*Yes*” vs. “0” = “*No*”). The sum score was obtained from all items to indicate IA. A total score ≥ 4 was classified as addicted to the Internet according to Young’s criteria. Previous research provided support for the psychometric properties of the measure (Shek et al., 2008; Shek and Yu, 2016).

##### 2.2.1.4. Trauma Screening Questionnaire

PTSD was gauged using Trauma Screening Questionnaire (TSQ) (Brewin et al., 2002). Participants reported whether they had experienced the specified post-traumatic symptoms “at least twice a week” under the COVID-19 on a dichotomous scale (“0” = “*have not experienced*” or “1” = “*have experienced*”). The sum of all item scores indicates the severity of PTSD symptoms. The psychometric properties of the measure were supported (Jiang et al., 2018).

##### 2.2.1.5. Scale of suicidal behavior

A three-item measure was used to evaluate suicidal behavior, which asked the participants whether they have had any plans, thoughts, and attempts related to suicide in the past 1 year (“1” = “*Yes*” and “0” = “*No*”) (Shek and Yu, 2012). The sum score of items was obtained as the scale score. The psychometric properties of the measure were supported by previous studies (alpha = 0.68–0.72) (Zhu and Shek, 2021).

##### 2.2.1.6. Hopelessness scale

Hopelessness was measured using a revised version of the “Beck Hopelessness Scale” (Beck et al., 1974; Shek, 1990). This measure includes five items regarding individuals’ levels of hopelessness about life. Each item was responded by the participants through a rating scale of six points (“1″ = “*Strongly disagree*” to “6″ = “*Strongly agree*”). The mean score of all items indicates hopelessness in this study. Previous studies supported the psychometric properties of the study (Shek, 1993).

As psychological morbidity was indicated by the composite scores of the above six mental health problems in the tested structural equation modelling (SEM) models, confirmatory factor analysis (CFA) was conducted to examine the validity of the latent variable. Different model fit indices were adopted to examine the fitness of CFA models, including “Comparative Fit Index (CFI),” “Tucker-Lewis Index (TLI),” “root mean square error of approximation (RMSEA)” and “standardized root mean squared residual (SRMR).” The CFI and TLI values >0.90 together with RMSEA and SRMR values ≤0.08 indicate acceptable model fit (Kline, 2011). The model fit indices indicated a good fit of the one-factor model: χ^2^/df = 7.347; CFI = 0.964; TLI = 0.932; RMSEA = 0.081; SRMR = 0.042. The factor loadings were also acceptable (ranging between 0.33 and 0.90).

#### 2.2.2. Positive well-being measures

##### 2.2.2.1. The Satisfaction with Life Scale

The present study used Satisfaction with Life Scale (SWLS) (five items) (Diener et al., 1985) to examine life satisfaction. The participants rated their agreement with each statement regarding their general satisfaction with life on a rating measure of six points from “1” = “*Strongly disagree*” to “6” = “*Strongly agree*.” The level of life satisfaction was represented by the item mean score. Previous research supported the psychometric properties of the scale (Sachs, 2003). To examine the psychometric properties of the scale in this study, CFA (one-factor model) was conducted. The results indicated a good model fit: χ^2^/df = 3.401; CFI = 0.997; TLI = 0.991; RMSEA = 0.050; SRMR = 0.010. The factor loadings of items ranged between 0.59 and 0.90.

##### 2.2.2.2. Flourishing Scale

Flourishing Scale (FS) was adopted to assess flourishing. FS has eight items measuring one’s psychological well-being in different life domains (e.g., life purpose, self-esteem, and psychological functioning) (Diener et al., 2010). The participants answered each item on a scale of seven points (“1” = “*Strongly disagree*” to “7” = “*Strongly agree*”). Flourishing is indicated by the mean score of all items. The good psychometric properties of the scale were supported by previous research (Tang et al., 2016). CFA based on a one-factor model was conducted to assess the psychometric properties of FS in this study, which indicated a good model fit: χ^2^/df = 9.523; CFI = 0.972; TLI = 0.957; RMSEA = 0.093; SRMR = 0.024, with factor loadings of all items ranging between 0.70 and 0.85.

#### 2.2.3. Measures of COVID-19 related factors

##### 2.2.3.1. COVID Stress Scale

As a risk factor, the COVID-19 related stress was evaluated by a modified version of the “COVID Stress Scale” (Taylor et al., 2020). This scale consists of three dimensions (each having five items), including “the danger and contamination of COVID-19,” “the socio-economic consequences of COVID-19,” and “check behavior because of concerns about COVID-19.” Participants reported the frequency of their experiencing the situation described by each item through a rating measure with five points (“0” = “*Not at all*” to “4” = “*Always*”). The mean score was gained to indicate COVID stress. CFA (three-factor model) was conducted for the scale in this study, which indicated a good model fit: χ^2^/df = 7.548; CFI = 0.944; TLI = 0.931; RMSEA = 0.082; SRMR = 0.053. Factor loadings ranged between 0.64 and 0.89.

##### 2.2.3.2. COVID-19 self-efficacy

As a protective factor, the COVID-19 related self-efficacy was assessed by a modified measure developed based on the “General Self-Efficacy Scale” (GSE) (Schwarzer and Jerusalem, 1995). The GSE contains 10 items assessing an individual’s positive self-beliefs on coping with different difficulties in life. It was widely used in different studies and demonstrated good psychometric properties (Luszczynska et al., 2005). In the present study, we have modified wordings of items in GSE to specifically assess the participants’ perceived self-efficacy in dealing with their life and study challenges during the COVID-19 pandemic. On each item, the participants rated to what extent the statement was true through a rating measure of five points (“1″ = “*Not at all*” to “6″ = “*Exactly true*”). The mean score was used as the indicator of COVID-19 self-efficacy. CFA (one-factor model) was conducted which indicated good model fit: χ^2^/df = 5.992; CFI = 0.967; TLI = 0.956; RMSEA = 0.071; SRMR = 0.027. The factor loadings ranged between 0.68 to 0.74.

#### 2.2.4. Measures of positive psychological attributes

##### 2.2.4.1. Chinese Cultural Beliefs about Adversity Scale

We adopted Cultural Beliefs about Adversity Scale (CBA) (Shek et al., 2003) to assess participants’ adversity-related beliefs. The scale includes nine items with each describing a traditional Chinese saying related to beliefs regarding adversity (seven items are on positive beliefs and two items are on negative beliefs). Each item is rated on a measure of six points (“1” = “*Strongly disagree*” to “6” = “*Strongly agree*”). The scale score was gained by averaging all item scores. The psychometric properties of the scale were supported by previous research (Leung and Shek, 2013). In our present study, we deleted the two reverse-code items due to the reason that the validity of reverse-coded items was challenged by different scholars (Autman and Kelly, 2017). CFA (one-factor model) indicated desirable model fit: χ^2^/df = 7.634; CFI = 0.966; TLI = 0.949; RMSEA = 0.082; SRMR = 0.027, with factor loadings ranging between 0.57 and 0.83.

##### 2.2.4.2. Chinese Positive Youth Development Scale (CPYDS).

Emotional competence and resilience were measured by two three-item subscales from the Chinese Positive Youth Development Scale (CPYDS) (Shek et al., 2007). The CPYDS assesses 15 positive youth development (PYD) constructs in Chinese adolescents based on the PYD constructs proposed by Catalano et al. (2002). Previous studies provide support to the psychometric properties of CPYDS (Shek et al., 2006, 2007). For the two subscales adopted in this study, the participants answered each item through a rating measure with six points (“1” = “*Strongly disagree*” to “6” = “*Strongly agree*”). The mean scores of the two subscales were obtained as the indicators of resilience and emotional competence, respectively. CFA was conducted for the two subscales, respectively. As each subscale contains only three items, the two CFA models were saturated models with factor loadings ranging between 0.64 and 0.87.

#### 2.2.5. Measures of environmental factors

##### 2.2.5.1. Chinese Family Assessment Instrument

The Chinese Family Assessment Instrument (C-FAI) is a locally developed measure assessing family process in terms of five dimensions (Shek, 2002), which demonstrated good psychometric properties in previous research (Siu and Shek, 2005). This study adopted the subscales of “Family Communication” (3 items) and “Family Mutuality” (3 items) in C-FAI to assess positive family functioning. The participants answered each item through a measure of five points (“1” = “*Very unlike*” to “5” = “*Very like*”). The composite score was indicated by the item mean score. CFA (one-factor model) was conducted, which showed good model fit: χ^2^/df = 4.354; CFI = 0.993; TLI = 0.985; RMSEA = 0.059; SRMR = 0.016. The factor loadings ranged between 0.55 and 0.85.

##### 2.2.5.2. Multidimensional Scale of Perceived Social Support

The Multidimensional Scale of Perceived Social Support (MSPSS) is a 16-item multidimensional scale assessing social support from three aspects including family, peers, and significant others (Zimet et al., 1988). The scale was validated based on samples in different cultural contexts including the context of Hong Kong (Chou, 2000). This study adopted the peer support subscale (four items) in MSPSS to measure perceived peer support. The participants indicated their agreement with each item through a five-point measure (“1” = “*strongly disagree*” to “5” = “*strongly agree*”). The item mean score was used to indicate participants’ perceived peer support. CFA (one-factor model) was conducted for the adopted peer support subscale, which indicated desirable model fit: χ^2^/df = 2.564; CFI = 0.999; TLI = 0.995; RMSEA = 0.040; SRMR = 0.004. The factor loadings ranged between 0.62 and 0.89.

##### 2.2.5.3. Community atmosphere measure

Community support was measured by three items selected from the Collective Efficacy Scale (CES) (Sampson et al., 1997). CES assesses social cohesion in neighborhoods in three dimensions including community cohesion, teacher social control and student social control which showed good psychometric properties in previous research (Peraza-Balderrama et al., 2021). The three items were selected from the community cohesion dimension which ask the participants to indicate to what extent they view their communities as caring and loving. Students offered their agreements with each item through a five-point measure (“1” = “*strongly disagree*” to “5” = “*strongly agree*”). The mean score of the three items was used to indicate participants’ perceived community support. As the measure contains three items, the CFA model was a saturated model with factor loadings ranging from 0.74 to 0.82.

#### 2.2.6. Need satisfaction, difficulties encountered, service usefulness and service evaluation

##### 2.2.6.1. Need satisfaction

A self-developed measure was adopted to assess students’ need satisfaction during the COVID-19, which can be regarded as a protective factor. The measure was developed by the research team of this study based on pilot focus group interviews conducted before the survey to collect information on students’ perceived needs in different domains under the pandemic. The measure contains 15 items asking students to rate the extent to which their needs have been satisfied in different domains under the pandemic, including physical, psychological, social, academic, and familial domains. Each item was responded on a measure of six points with “1″ representing “*not met at all*” and “6″ representing “*fully met*.” A mean score was gained as the scale score. The measure showed good reliability in previous research (alpha = 0.89) (Shek et al., 2022b). A one factor model of CFA was conducted to examine the psychometric properties of the scale, with the results indicating acceptable model fit: χ^2^/df = 7.661; CFI = 0.909; TLI = 0.883; RMSEA = 0.083; SRMR = 0.049. The factor loadings ranged between 0.41 and 0.69.

##### 2.2.6.2. Difficulties encountered

A measure with 24 items was developed to assess difficulties and challenges students may encounter in different domains during the COVID-19 pandemic. On each item, the participants indicated to what degree they encountered the specified difficulty through a measure of five points with “1” being “*Never*” and “5” being “*Always*.” An average score was gained as the total score. Previous research showed good internal consistency of the scale (Shek et al., 2022a). Results of CFA (one factor model) indicated acceptable model fit: χ^2^/df = 4.647; CFI = 0.903; TLI = 0.887; RMSEA = 0.061; SRMR = 0.053, with factor loadings ranging between 0.44 and 0.67.

##### 2.2.6.3. Perceived usefulness of university service

A measure with 10 items was developed by the research team of this study to assess students’ perceived usefulness of the university services related to the COVID-19. These services include “Counselling and Wellness Section,” “University Health Service,” “Special Funding under COVID-19″, etc. Students needed to rate their perceived usefulness of these services through a measure of six points with “1″ being “*Not at all*” and “6″ being “*Completely yes*.” A mean score was gained as the total score. CFA (one factor model) was conducted for the measure, with results indicating acceptable model fit: χ^2^/df = 3.339; CFI = 0.911; TLI = 0.866; RMSEA = 0.074; SRMR = 0.051, although two items have slightly lower loadings.

##### 2.2.6.4. Evaluation of university service

Another measure with 12 items was developed by the research team of this study to assess students’ satisfaction with the services provided by the university during the COVID-19. Through a six-point rating scale, the students needed to indicate their levels of satisfaction with university service/support in different domains such as teaching and learning, counselling, campus facilities, etc. A mean score was gained as the total score. The results of CFA indicated good model fit: χ^2^/df = 8.278; CFI = 0.959; TLI = 0.945; RMSEA = 0.086; SRMR = 0.030. The factor loadings ranged between 0.72 and 0.84.

### 2.3. Data analyses

Descriptive and reliability analyses were conducted for all variables. We first investigated the profiles of psychological morbidity (negative mental health) among the participants, particularly those scales with cutoff scores (i.e., DASS-21, CESD-R, and PTSD symptoms). Then, Pearson’s correlation coefficients were computed to show inter-correlations among different risk and protective factors and the outcome variables (i.e., psychological morbidity, life satisfaction, and flourishing). Finally, SEM was run to examine the predictive effects of the COVID-19 related factors (Model 1), positive psychological attributes (Model 2), environmental factors (Model 3), and need satisfaction, difficulties, service usefulness, and service evaluation (Model 4) on psychological morbidity, life satisfaction, and flourishing. The analyses were performed using SPSS (Version 26) and Mplus (Version 8.3).

We used G*Power to perform power analysis of the study based on *R*^2^ increase in the SEM models. For each SEM model, the Mplus results reported the *R*^2^ values indicating the proportion of variance explained by the predictors. Based on the *R*^2^ values (ranging between 0.09 and 0.27), the estimated sample size for power = 0.09 for the predictors in the four SEM models ranged between 43 and 161. This showed that our sample size of 978 ensured sufficient statistical power to detect the effect of predictors.

## 3. Results

### 3.1. Descriptive statistics and reliability of the measures

The descriptive and reliability statistics are presented in Table 2. All measures in this study demonstrated acceptable reliability (α ranged between 0.65 and 0.96), and inter-item correlations were between 0.27 and 0.70.

### 3.2. Prevalence of psychological morbidity (mental health problems)

Table 3 presents the prevalence rates and profile of mental health problems among the sample. For DASS-21, 53.9, 61.3, and 36.5% of the students were classified as having mild-to-above levels of depression, anxiety, and stress, respectively. For CESD-R, 41.2% of the students were classified as having clinically significant depressive symptoms according to Radloff’s criteria (scored 16 or above) (Radloff, 1977). Regarding PTSD, 21.3% of the students met the screening criteria for PTSD (i.e., obtaining a score of 6 or higher in TSQ) (Brewin et al., 2002). Finally, for Internet addiction, over three quarters of the participants (78.4%) were identified as showing Internet addiction problems (i.e., reporting four or more symptoms) (Shek et al., 2008). Regarding suicidal behavior, 12.2% of the students reported having suicidal thoughts, but only small proportions of the students had suicidal plans (4.4%) and suicidal attempts (3.1%).

**Table 3:** Prevalence of negative mental health.

Measure and category	*n*	%
DASS-21-Depression^a^		
Normal	450	46.0
Mild	146	14.9
Moderate	242	24.7
Severe	101	10.3
Extremely severe	39	4.0
DASS-21-Anxiety^b^		
Normal	379	38.8
Mild	90	9.2
Moderate	234	23.9
Severe	117	12.0
Extremely Severe	158	16.2
DASS-21-Stress^c^		
Normal	621	63.5
Mild	147	15.0
Moderate	125	12.8
Severe	78	8.0
Extremely Severe	7	0.7
CESD-R		
With symptoms (scored 16 or above)	403	41.2
Without symptoms	575	58.8
TSQ		
Met criteria (scored 6 or above)	208	21.3
Not met	770	78.7
IAT-10		
Addicted (scored 4 or above)	767	78.4
Not addicted	211	21.6

^a^Depression: Normal = 0–9, Mild = 10–13, Moderate = 14–20, Severe = 21–27, Extremely severe = 28 or above. ^b^Anxiety: Normal = 0–7, Mild = 8–9, Moderate = 10–14, Severe = 15–19, Extremely severe = 20 or above. ^c^Stress: Normal = 0–14, Mild = 15–18, Moderate = 19–25, Severe = 26–33, Extremely severe = 34 or above.

### 3.3. Sociodemographic predictors

Multiple regression analyses were conducted to examine the predictive effects of different sociodemographic factors (Table 4). Year of study negatively predicted CESD-R and Internet addiction (*β* = −0.11 and – 0.09, *p* < 0.05 and = 0.05, respectively). In addition, while living status did not predict mental health problems, it negatively predicted life satisfaction and flourishing (*β* = −0.08 and – 0.14, *p* < 0.05 and *p* < 0.001). With regard to economic factors, CSSA positively predicted CESD-R (*β* = 0.10, *p* < 0.01) but did not predict other mental health problems and well-being indicators; having family financial difficulty positively predicted DASS-Depression and PTSD (*β* = 0.11, *p* < 0.05) but did not predict other indicators; having personal financial difficulty positively predicted Internet addiction (*β* = 0.12, *p* < 0.05) while negatively predicted life satisfaction (*β* = −0.13, *p* < 0.01); personal or family members’ unemployment positively predicted DASS-Stress, CESD-R, and Internet addiction (*β* = 0.09–0.11, *p* < 0.05 and *p* < 0.01, respectively). The results supported Hypothesis 1a and Hypothesis 1b.

**Table 4:** Predicting effects of sociodemographic factors on mental health and well-being.

Sociodemographic predictors^a^												
	1	2	3	4	5	6	7	8	9	10	11	12
Negative mental health												
DASS-21												
Depression	0.08	−0.01	−0.02	−0.07	0.05	0.07	0.11*	0.08	0.07	−0.05	0.02	0.01
Anxiety	0.01	−0.04	−0.04	−0.04	0.04	0.05	0.09	0.08	0.06	−0.06	0.03	−0.02
Stress	0.07	−0.04	−0.06	−0.05	0.03	0.06	0.09	0.10	0.09*	−0.01	0.01	0.04
PTSD	0.08	0.03	−0.08	0.07	0.05	−0.01	0.11*	0.03	0.05	0.02	−0.02	−0.04
CESD-R	0.11*	−0.02	−0.11*	−0.06	0.07	0.10**	0.09	0.06	0.10**	−0.13**	0.05	−0.01
Suicidal behavior	0.04	0.01	−0.08	0.08	−0.01	−0.01	0.09	0.04	−0.04	0.02	−0.10*	−0.00
Internet addiction	0.05	0.05	–0.09^a^	0.03	0.00	−0.04	0.07	0.12*	0.11**	−0.01	−0.01	0.05
Hopelessness	−0.11*	0.02	0.07	−0.13**	−0.02	0.00	0.01	0.04	0.00	0.00	−0.05	−0.02
Positive well-being												
Life satisfaction	−0.06	−0.01	0.03	0.02	−0.08*	0.06	−0.08	−0.13**	−0.01	0.00	−0.01	0.03
Flourishing	−0.11*	0.04	0.07	0.00	−0.14***	0.02	−0.08	−0.07	−0.00	0.03	−0.02	0.10*

^a^Sociodemographic factors: 1 = Age; 2 = Gender (1 = male, 2 = female); 3 = Year of study; 4 = Place of origin (1 = local, 2 = international); 5 = Living status (0 = living with family or living with roommates, 1 = living alone); 6 = Received CSSA (0 = no, 1 = yes); 7 = Having family financial difficulty (0 = no, 1 = yes); 8 = Having personal financial difficulty (0 = no, 1 = yes); 9 = Family/personal unemployed (0 = no, 1 = yes); 10 = Personal/family members being confirmed case(s) of COVID-19 (0 = no, 1 = yes); 11 = Place of residence under the pandemic (1 = Hong Kong, 2 = mainland China, 3 = others). DASS-21, total score of depression, anxiety and stress scale; PTSD, post-traumatic stress disorder; CESD-R, The Center for Epidemiologic Studies Depression Scale Revised. ***p < 0.001; **p < 0.01; *p < 0.05; ^a^*p* = 0.05.

### 3.4. Predictive effects of different psychosocial risk and protective factors

Table 5 shows the inter-correlations among the hypothesized risk and protective variables and mental health outcome variables.

**Table 5:** Correlations among key variables.

	1	2	3	4	5	6	7	8	9	10	11	12	13	14	15	16	17	18	19	20	21
1. C19S _DC	--																				
2. C19S _SC	0.52^***^	--																			
3. C19S _CB	0.62^***^	0.67^***^	--																		
4. SE	−0.06	−0.13^***^	−0.02	--																	
5. DASS-21	0.25^***^	0.47^***^	0.36^***^	−0.19^***^	--																
6. PTSD	0.32^***^	0.37^***^	0.35^***^	−0.19^***^	0.42^***^	--															
7. CESD-R	0.27^***^	0.53^***^	0.41^***^	−0.18^***^	0.80^***^	0.41^***^	--														
8. SB	0.09^**^	0.12^***^	0.13^***^	−0.05	0.27^***^	0.25^***^	0.29^***^	--													
9. IAT	0.26^***^	0.25^***^	0.27^***^	−0.09^**^	0.39^***^	0.46^***^	0.37^***^	0.34^***^	--												
10. HL	0.07^*^	0.17^***^	0.10^**^	−0.23^***^	0.35^***^	0.15^***^	0.34^***^	0.12^***^	0.18^***^	--											
11. LS	−0.11^***^	−0.05	−0.03	0.22^***^	−0.26^***^	−0.19^***^	−0.27^***^	−0.16^***^	−0.24^***^	−0.22^***^	--										
12. FL	−0.07^*^	−0.18^***^	−0.10^**^	0.36^***^	−0.47^***^	−0.23^***^	−0.49^***^	−0.21^***^	−0.22^***^	−0.39^***^	0.65^***^	--									
13. BA	−0.02	−0.20^***^	−0.10^**^	0.18^***^	−0.36^***^	−0.16^***^	−0.40^***^	−0.13^***^	−0.14^***^	−0.28^***^	0.37^***^	0.52^***^	--								
14. RE	−0.06^*^	−0.15^***^	−0.08^*^	0.26^***^	−0.37^***^	−0.15^***^	−0.40^***^	−0.17^***^	−0.13^***^	−0.29^***^	0.39^***^	0.63^***^	0.53^***^	--							
15. EC	−0.07	−0.24^***^	−0.18^***^	0.25^***^	−0.44^***^	−0.20^***^	−0.48^***^	−0.16^***^	−0.18^***^	−0.24^***^	0.34^***^	0.59^***^	0.51^***^	0.70^***^	--						
16. PFAM	−0.05	−0.18^***^	−0.09^**^	0.16^***^	−0.35^***^	−0.16^***^	−0.34^***^	−0.18^***^	−0.18^***^	−0.20^***^	0.37^***^	0.45^***^	0.36^***^	0.39^***^	0.41^***^	--					
17. PS	−0.09^**^	−0.29^***^	−0.19^***^	0.24^***^	−0.38^***^	−0.21^***^	−0.41^***^	−0.11^***^	−0.13^***^	−0.20^***^	0.29^***^	0.55^***^	0.43^***^	0.45^***^	0.47^***^	0.40^***^	--				
18. CA	−0.12^***^	−0.14^***^	−0.11^**^	0.06	−0.27^***^	−0.11^**^	−0.25^***^	−0.11^**^	−0.17^***^	−0.12^***^	0.32^***^	0.37^***^	0.34^***^	0.32^***^	0.35^***^	0.43^***^	0.36^***^				
19. NEED	−0.14^***^	−0.17^***^	−0.11^***^	0.24^***^	−0.35^***^	−0.24^***^	−0.39^***^	−0.13^***^	−0.24^***^	−0.22^***^	0.47^***^	0.56^***^	0.44^***^	0.44^***^	0.45^***^	0.46^***^	0.42^***^	0.43^***^			
20. DIF	0.37^***^	0.34^***^	0.34^***^	−0.18^***^	0.40^***^	0.31^***^	0.35^***^	0.11^**^	0.36^***^	0.24^***^	−0.25^***^	−0.21^***^	−0.10^**^	−0.07^*^	−0.11^***^	−0.15^***^	−0.07^**^	−0.14^***^	−0.26^***^		
21. US	0.02	−0.13^***^	−0.07^*^	0.13^***^	−0.19^***^	−0.12^***^	−0.22^***^	−0.08^*^	−0.07^*^	−0.12^**^	0.14^***^	0.25^***^	0.23^***^	0.27^***^	0.29^***^	0.18^***^	0.32^***^	0.15^***^	0.31^***^	−0.05	
22. ES	−0.08^*^	−0.23^***^	−0.16^***^	0.26^***^	−0.28^***^	−0.19^***^	−0.34^***^	−0.07^*^	−0.12^***^	−0.16^***^	0.30^***^	0.39^***^	0.43^***^	0.39^***^	0.44^***^	0.28^***^	0.35^***^	0.27^***^	0.49^***^	−0.16^***^	0.45^***^

C19S, COVID-19 related stress; DC, anger and contamination; SC, socio-economic consequences; CB, check behavior; SE, self-efficacy related to COVID-19; DASS-21, total score of depression, anxiety stress scale; DASS_D, depression, DASS_A, anxiety; DASS_S, stress; PTSD, post-traumatic stress disorder; CESD-R, The Center for Epidemiologic Studies Depression Scale Revised; SB, suicidal behavior; IAT, internet addiction; HL, hopelessness; LS, life satisfaction; FL, flourishing; BA, beliefs of adversity; RE, resilience; EC, emotional competence; PFAM, positive family functioning; PS, peer support; CA, community atmosphere; NEED, need satisfaction; DIF, difficulties encountered; US, perceived usefulness of university services; ES, evaluation of university services. ***p < 0.001; **p < 0.01; *p < 0.05.

SEM was conducted to test the predictive effects of COVID-19 related risk and protective factors (Model 1), positive psychological attributes (Model 2), environmental factors (Model 3), and need satisfaction, difficulties encountered, service usefulness and service evaluation (Model 4) on negative mental health and well-being factors (life satisfaction and flourishing). Table 6 presents the model fit indices of the four models. All models fitted the data well, with CFI and TLI being above 0.90, RMSEA being below 0.08, and SRMR equaling to 0.05.

**Table 6:** Model fit of structural equation models.

Model	*χ^2^*/*df*	CFI	TLI	RMSEA	SRMR
Model 1: COVID-19 related factors - > outcomes	3.28	0.91	0.90	0.05	0.05
Model 2: Positive psychological attributes - > outcomes	3.45	0.92	0.91	0.05	0.05
Model 3: Positive environmental factors - > outcomes	4.26	0.92	0.92	0.06	0.05
Model 4: Needs, difficulties, and service evaluation - > outcomes	5.01	0.92	0.90	0.07	0.05

**Figure 5:**
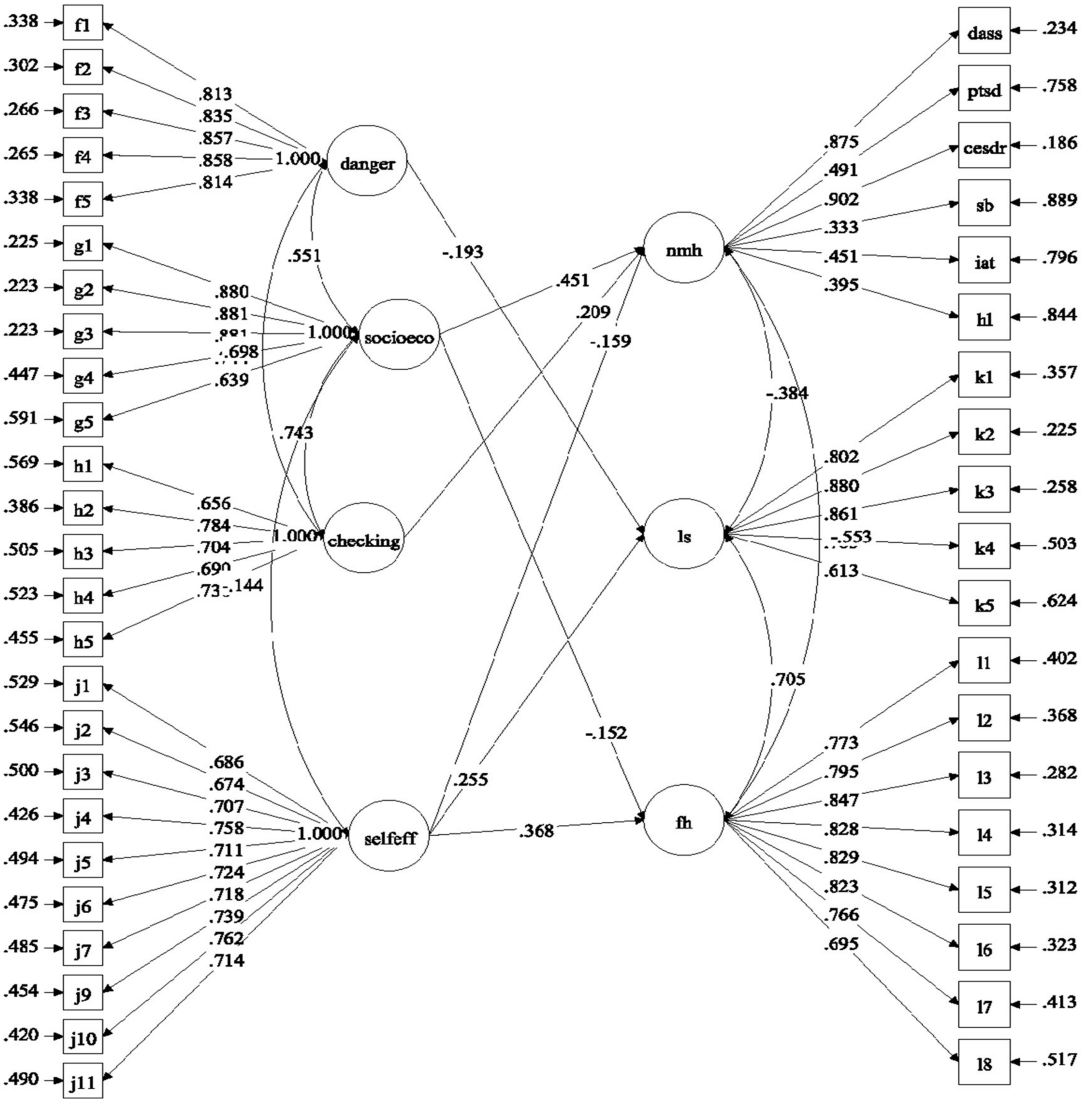
Predictive effects of COVID-19 related factors (Model 1). Standardized estimates were shown in the figure; danger, danger and contamination; socioeco, socio-economic consequences; checking, checking behavior; selfeff, self-efficacy related to COVID-19; nmh, negative mental health; Is, life satisfaction; fh, flourishing.

Table 7 shows the predictive effects of different risk and protective factors in different models. Regarding COVID-19 related factors (Model 1), the predictive effects of the three subscales of the pandemic related stress varied for the three outcome variables. Specifically, danger and contamination predicted life satisfaction in a negative direction (*β* = −0.19, *p* < 0.001). Higher socio-economic consequences predicted higher psychological morbidity (*β* = 0.45, *p* < 0.001) and lower flourishing (*β* = −0.15, *p* < 0.01). Checking behavior positively predicted psychological morbidity (*β* = 0.21, *p* < 0.01). Therefore, Hypothesis 2a and 2b were partially supported. For self-efficacy related to COVID-19, it predicted lower psychological morbidity (*β* = −0.16, *p* < 0.01) and higher life satisfaction and flourishing (*β* = 0.26 and 0.37, *p* < 0.001). This supports Hypothesis 2c and 2d. The predictive effects are shown in Figure 5.

**Table 7:** Predictive effects of COVID-19 related factors, positive psychological attributes, positive environmental factors, and need satisfaction, service evaluation, and difficulties on mental health.

Predictors	Outcomes					
	Negative mental health		Life satisfaction		Flourishing	
	*β*	*SE*	*β*	*SE*	*β*	*SE*
COVID-19 related factors (Model 1)						
Danger and contamination	−0.08	0.05	−0.19^***^	0.05	0.05	0.06
Socio-economic consequences	0.45^***^	0.05	0.00	0.06	−0.15^**^	0.05
Checking behavior	0.21^**^	0.07	0.11	0.08	−0.03	0.07
Self-efficacy related to COVID-19	−0.16^***^	0.03	0.26^***^	0.04	0.37^***^	0.04
Positive psychological attributes (Model 2)						
Beliefs of adversity	−0.16^**^	0.05	0.16^**^	0.06	0.15^**^	0.05
Resilience	0.07	0.10	0.27^**^	0.10	0.42^***^	0.08
Emotional competence	−0.53^***^	0.10	0.09	0.10	0.23^**^	0.08
Positive environmental factors (Model 3)						
Positive family functioning	−0.22^***^	0.04	0.23^***^	0.04	0.22^***^	0.03
Peer support	−0.35^***^	0.04	0.14^***^	0.04	0.44^***^	0.03
Positive community atmosphere	−0.08^ms^	0.04	0.20^***^	0.04	0.14^***^	0.04
Need satisfaction, service evaluation, and difficulties (Model 4)						
Need satisfaction	−0.27^***^	0.04	0.44^***^	0.03	0.49^***^	0.03
Difficulties encountered	0.34^***^	0.03	−0.13^***^	0.03	−0.07^*^	0.03
Perceived usefulness of services	−0.09^**^	0.03	−0.01	0.04	0.07^*^	0.03
Evaluation of services	−0.09^*^	0.04	0.06	0.04	0.08^*^	0.04

Negative mental health was indicated by depression, anxiety, stress (DASS), posttraumatic disorder symptoms, depressive symptoms (CESD-R), suicidal behavior, internet addiction, and hopelessness; ms, marginal significance (*p* = 0.058). ***p < 0.001; **p < 0.01; *p < 0.05.

**Figure 6:**
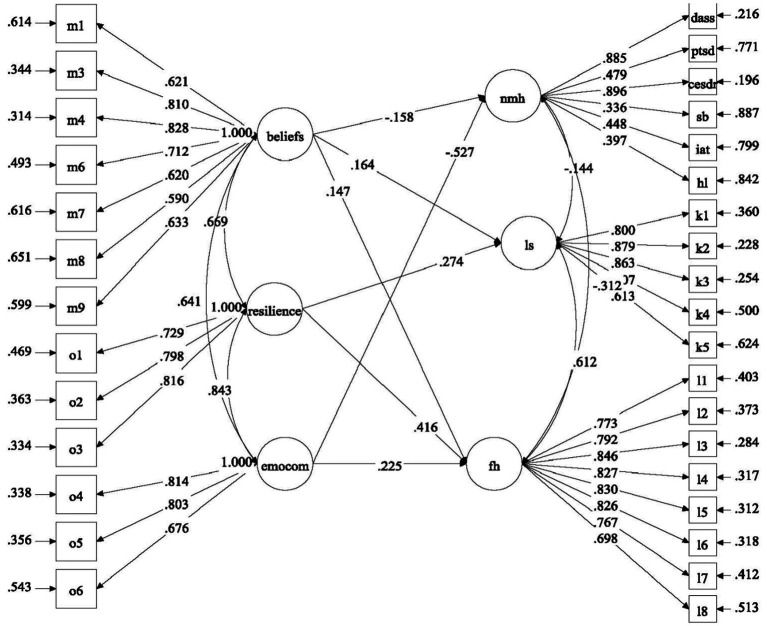
Predictive effects of positive psychological attributes (Model 2). Standardized estimates were shown in the figure; beliefs, beliefs of adversity; emotcom, emotional competence; nmh, negative mental health; ls, life satisfaction; fh, flourishing.

Regarding positive psychological attributes (Model 2, see Figure 6), most intrapersonal factors significantly predicted the outcome variables. Beliefs of adversity and emotional competence (*β* = −0.16 and − 0.53, *p* < 0.01 and *p* < 0.001, respectively) predicted lower psychological morbidity while resilience did not. Hypothesis 3a was partially supported. Besides, beliefs of adversity and resilience (*β* = 0.15–0.42, *p* < 0.01) predicted life satisfaction and flourishing in positive direction while emotional competence only predicted flourishing (*β* = 0.23, *p* < 0.01). Therefore, Hypothesis 3b was partially supported.

**Figure 7:**
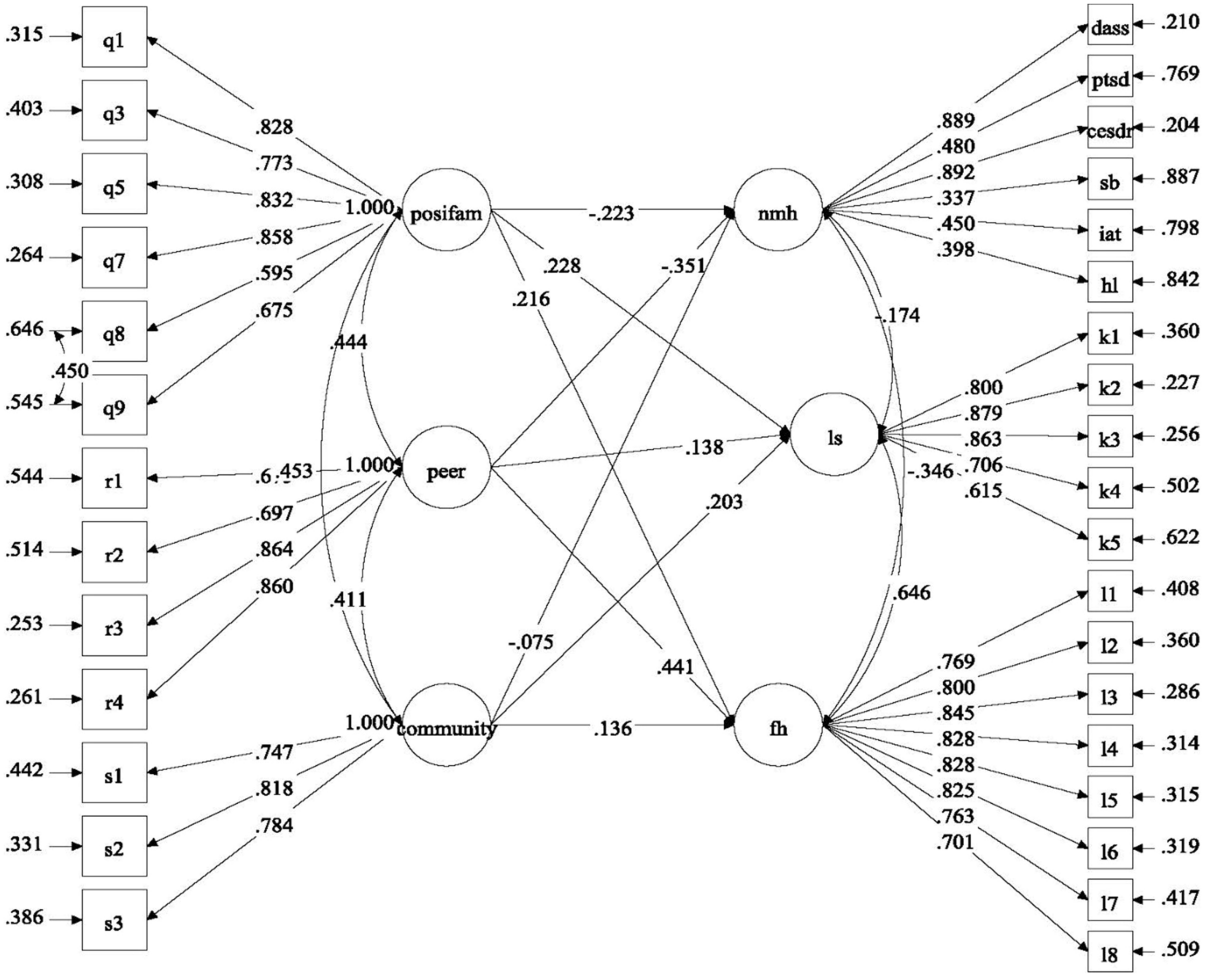
Predictive effects of positive extra-personal attributes (Model 3). Standardized estimates were shown in the figure; posifam, positive family functioning; peer, peer support; community, community support; nmh, negative mental health; ls, life satisfaction; fh, flourishing.

All environmental factors significantly predicted outcome variables (Model 3, see Figure 7). In specific, family functioning, peer support, and community atmosphere (*β* = −0.35 to −0.08, *p* < 0.001 and *p* = 0.058, respectively) negatively predicted psychological morbidity, which supported Hypothesis 4a. In addition, these factors all predicted flourishing and life satisfaction in positive direction (*β* = 0.14 to 0.44, *p* < 0.001), which supported Hypothesis 4b.

**Figure 8:**
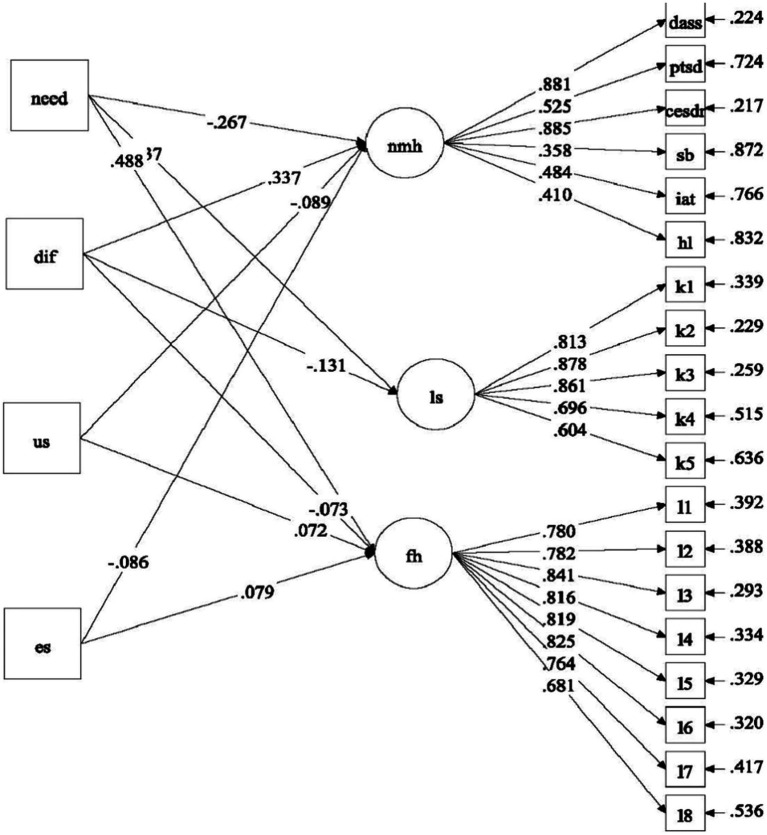
Predictive effects of need satisfaction, difficulties encountered, perceived usefulness of services and evaluation of services (Model 4). Standardized estimates were shown in the figure; need, need satisfaction; dif, difficulties encountered; us, perceived usefulness of services; es, evaluation of services; ls, life satisfaction; fh, flourishing.

For Model 4 (see Figure 8), while need satisfaction, perceived usefulness of services, and evaluation of services had negative predictive effects on psychological morbidity (*β* between −0.27 and −0.09, *p* < 0.05), difficulties encountered displayed a positive effect (*β* = 0.34, *p* < 0.001), supporting Hypothesis 5a. Besides, need satisfaction positively predicted two well-being indicators (*β* = 0.44 and 0.49, *p* < 0.001) and perceived usefulness of services and evaluation of services only positively predicted flourishing (*β* = 0.07 and 0.08, *p* < 0.05), while difficulties encountered negatively predicted two well-being indicators (*β* = −0.13 and − 0.07, *p* < 0.05). Hypothesis 5b was partially supported.

## 4. Discussion

The present study examined the mental health status of university students in Hong Kong under the COVID-19 pandemic. It has significant contribution to the existing research in this area. Although existing research has identified university students as a vulnerable group under the pandemic and attempted to understand related factors to reduce potential mental health problems, some research gaps need to be filled. First, while a substantial body of research on mental health in university students is conducted in the Western context, few studies have been conducted in the Hong Kong context. Due to the unique cultural and social contexts, such as socio-cultural risk factors (Shek and Siu, 2019), mental health in Hong Kong university students calls for further investigation. Second, there is limited literature on the psychosocial protective and risk factors of mental health in university students under the pandemic. Third, as many studies have focused only on limited risk and/or protective factors (Sun et al., 2020; Faisal et al., 2022), it would be theoretically illuminating to incorporate more ecological risk and protective factors in a single study. Fourth, there is limited research on the practical issues facing university students, such as need satisfaction, difficulties encountered, and perceived university services in relation to students’ mental health under the pandemic. To address the identified research gaps, this study provides a comprehensive investigation of mental health problems in university students in Hong Kong under the pandemic.

### 4.1. Prevalence of mental health problems in Hong Kong university students

This study revealed a high prevalence of mental health problems among university students in Hong Kong under the pandemic, with over half of them experiencing symptoms of depression and anxiety, and a sizeable proportion showing symptoms of stress, PTSD, and Internet addiction. These findings are consistent with previous research indicating that university students are vulnerable to negative mental health outcomes under the pandemic (Dragioti et al., 2022; Shek et al., in press; Yıldırım and Şanlı, 2023).

Compared to a previous study conducted before the pandemic (Leung, 2017), more university students in Hong Kong experienced moderate or above levels of depression (39% vs. 21%) and anxiety (52.1% vs. 41%) in the present study. Furthermore, the prevalence of mental health problems among university students in Hong Kong was similar to those based on Western countries or exceeded those of some Asian regions during the outbreak of the pandemic. For example, in the United Kingdom, more than 50% of university students showed clinically significant depression and anxiety under the pandemic (Chen and Lucock, 2022). In a study among 2,031 American university students, 38.5% reported a moderate-to-severe level of anxiety, and 48.1% had a moderate-to-severe level of depression (Wang et al., 2020). In Singapore, according to a survey of 1,779 university students, the prevalence of depression and anxiety symptoms was high at 32 and 25%, respectively (Yong and Keh, 2022). Besides, the present study found a particularly high prevalence of Internet addiction (around 78%). This is higher than the prevalence rate (around 50%) reported in a study conducted in the 4^th^ wave of the pandemic (Shek et al., 2023a). As currently quite limited research was done on Internet addiction prevalence in tertiary students under the pandemic, the present study sheds light on the issue which indicates that more attention should be paid to the prevention of Internet addition in university students under the pandemic (Shek et al., 2023a).

### 4.2. Sociodemographic and COVID-19 related predictors of psychological well-being

Consistent with the existing literature (Nadareishvili et al., 2022; Galanza et al., 2023), results of this study revealed that having financial problems under the pandemic was linked to higher psychological morbidity and lower well-being. For living status, compared with living with family members and roommates, living alone predicted lower life satisfaction and flourishing. This is also in line with some existing research (Husky et al., 2020). Therefore, special attention should be paid to these groups of students. However, the study did not find predictive effects of gender, which was not in line with many existing studies (Patsali et al., 2020; Prowse et al., 2021). Hence, the association between gender and mental health in tertiary students in the pandemic period should be further explored.

For COVID-19 stress, two subscales of COVID-19 related stress (i.e., socioeconomic consequence and checking behavior) positively predicted negative mental health. This is consistent with findings from some existing studies which showed positive association between the two dimensions of COVID-19 stress and psychological morbidity such as anxiety, depression, and PTSD (Adamczyk et al., 2021; Jungmann et al., 2023). However, as most of these studies were conducted based on community or adult samples, the present study is pioneer in nature. Interestingly, the danger and contamination dimension did not predict negative mental health, which was not in line with existing literature (Adamczyk et al., 2021; Jungmann et al., 2023). This may possibly be due to the fact that Hong Kong had a very stringent health policy under COVID-19 (Chan H. Y. et al., 2021). In addition to the mask mandate and social distancing measures, the “StayHomeSafe” scheme was launched on 8 February 2022 as a cost-effective alternative for infected persons and their close contacts. Infected individuals and their close contacts have to quarantine themselves at home for 14 days and 4 days, respectively, depending on a quick assessment (Du et al., 2022). Furthermore, the Hong Kong government implemented a “dynamic zero infection” policy to combat the pandemic. Under this policy, the Hong Kong government has adopted the “test-trace-isolate-quarantine” (TTIQ) strategy to prevent the spread of the disease, which involves isolating confirmed patients from the community and tracing and identifying close contacts (Lau et al., 2022). Another possibility is that as COVID-19 affects people who are old and/or having chronic diseases, related dangers are not related to psychological well-being in university students who are relatively young.

Besides, results of this study showed that pandemic related self-efficacy negatively predicted mental health problems and positively predicted well-being, which is in line with the findings from existing limited research (Sun et al., 2021). This indicates that pandemic related self-efficacy is a strong protective factor for psychological well-being of college students in the pandemic period. This is consistent with the observation that general self-efficacy is an important developmental asset in adolescent development (Shek et al., 2015).

### 4.3. Psychosocial predictors of psychological well-being

Results of this study identified that emotional competence negatively predicted mental health problems while positively predicted flourishing. This provides additional support to the extant literature (Li N. et al., 2021; Krifa et al., 2022), indicating a protective function of emotional competence in psychological well-being of university students in the pandemic period. However, resilience only predicted positive well-being indicators but not psychological morbidity in the present study, which is not in accordance with the existing literature (Chi et al., 2020; Alyoubi et al., 2021; Yıldırım and Çiçek, 2022; Yıldırım and Ashraf, 2023). The present study also sheds light on the protective role of adversity related beliefs in psychological well-being of college students under the pandemic. While there is a body of research indicating the protective role of adversity-related beliefs in Chinese culture in psychological functioning of Hong Kong adolescents (Shek, 2004, 2005), whether these beliefs would also protect university students against negative influence of the pandemic has not been investigated. Findings from this study fill in this research gap.

For the environmental factors, the present study showed that positive family functioning, peer support, and supportive community atmosphere all negatively predicted mental health problems and positively predicted well-being factors which is consistent with the extant literature (Sun et al., 2020; Nadareishvili et al., 2022). The results advance the existing literature as there is limited research in the context of Hong Kong. Besides, as pointed out by Shek et al. (2023c), most of the existing policies combating COVID-19 are financial or medical in nature, with very little attention paid to the importance of psychosocial support under the pandemic.

Finally, the study contributes to the extant literature showing that need satisfaction and positive evaluation of university services negatively predicted psychological morbidity and positively predicted well-being. One reason for this relationship may be that students rely on universities to provide them with support and resources during this stressful time, and if they perceive those services as inadequate or ineffective, they may feel unsupported and isolated (Lipson et al., 2019). This can exacerbate feelings of stress, anxiety, and depression, and can lead to a sense of disengagement from academic and social life (Arnett et al., 2014). The results also showed that students who perceived more difficulties during the pandemic reported higher levels of mental health problems and lower well-being. The finding is in line with the existing few empirical studies. One study revealed that need satisfaction predicted lower depressive symptoms in college students under the pandemic (Shek et al., 2022b) and another study found that higher difficulties in academic study and employment were linked to higher depression under the pandemic (Kecojevic et al., 2020). As there are not many studies in this area, these are novel findings in the COVID-19 literature.

## 5. Implications, limitations, and conclusion

The study has theoretical implications. As for theoretical aspect, the integration of the ecological systems approach and the strength-based perspective is essential for comprehending the intricate interplay of factors affecting university students’ mental health. This combined approach facilitates a more comprehensive and nuanced understanding of the diverse elements that contribute to students’ well-being and resilience. By adopting this framework, results of this study strengthen the argument that various factors within the ecological system play risk and protective roles in shaping the mental health of university students, with a particular emphasis on personal factors as well as the influence of family, peers, and the community (Shek et al., 2023b). In addition, the study is consistent with strength-based perspectives, which highlight the importance of internal and external assets in promoting healthy development among adolescents (Benson, 2007). Specifically, the study emphasizes the importance of cultural beliefs regarding adversity in determining the psychological well-being of college students during the COVID-19 pandemic. Given that cultural beliefs are strongly influenced by social cultures, norms, and values within the macrosystem, this research addresses the need for more indigenous studies conducted in Chinese contexts (Shek et al., 2022a,b). These findings are consistent with Liu et al. (2017) assertion that a multi-level, dynamic, and integrative model of adversity coping should consider socio-ecological, interpersonal, and individual factors.

For practical implication, the study provides important research-based evidence for better intervention and prevention of mental health symptoms in tertiary students. It points to the importance of paying special attention to the mental health status of students with financial or economic difficulties. It also highlights the direction of promoting intrapersonal and ecological protective factors and reducing risk factors in the treatment and intervention programs. Furthermore, the study highlights the important role of attending to need satisfaction and difficulties faced by university students. Also, helping students to gain a positive perception of the usefulness and value of the services received from the university is important.

Despite its pioneer nature, this study has several limitations. First, while the study adopted a quota sampling method (as it is difficult to do random sampling under the pandemic), future studies might be conducted based on random sampling to replicate the findings. However, quota sampling is commonly adopted in studies under COVID-19 (McBride et al., 2021; Futri et al., 2022). Second, as the study adopted a cross-sectional design, longitudinal studies are needed in future to further examine the roles of different risk and protective factors. Third, as the participants of this study came from one university which is not uncommon under the pandemic (e.g., Kecojevic et al., 2020; Chen and Lucock, 2022), students from multiple universities should be involved in future research. Fourth, as we adopted English version of measures in this study, future research should be conducted to use translated and/or indigenously developed measures for Chinese students. Despite of the limitations, the study contributes to exiting limited literature on mental health of tertiary students under the pandemic in Hong Kong, which provides important directions for future research and implications for intervention and university service improvement.

To conclude, the present study examined mental health of university students in Hong Kong (indexed by psychological morbidity and positive well-being) under the pandemic and the related risk and protective factors, including financial difficulties encountered, COVID-19 stress and responses, positive psychological attributes, environmental factors and needs and services factors. The present study suggests that pandemic related stress and perceived difficulties are important risk factors whereas positive psychological and environmental attributes are protective factors. It also suggests the positive role of need satisfaction, perceived usefulness, and satisfaction with university services in the mental health of university students. It enriches the existing literature on the mental health of university students under the pandemic by contributing to our understanding of the predictors of mental health in university students and providing important pointers for prevention and intervention.

## Data availability statement

The raw data supporting the conclusions of this article will be made available by the authors, without undue reservation.

## Ethics statement

The studies involving human participants were reviewed and approved by Institutional Review Board (or its Delegate) at the Hong Kong Polytechnic University. The patients/participants provided their written informed consent to participate in this study.

## Author contributions

DS obtained the research grant, conceived the research, contributed to all stages of the research work, and critically revised all versions of the manuscript. WC conducted the data analyses, drafted the manuscript, and conducted the revision and checking. XL revised the discussion section and checked the whole manuscript. DD revised the introduction section of the manuscript.

## Funding

This project was financially supported by a UGC special grant for student support services in response to the COVID-19 pandemic entitled “Promotion of psychological well-being in university students under COVID-19: Needs assessment and mental health survey” (project no.: 89S7). The publication of this paper was financially supported by the Research Grants Council Matching Fund and Wofoo Foundation (R-ZH4Q).

## Conflict of interest

The authors declare that the research was conducted in the absence of any commercial or financial relationships that could be construed as a potential conflict of interest.

## Publisher’s note

All claims expressed in this article are solely those of the authors and do not necessarily represent those of their affiliated organizations, or those of the publisher, the editors and the reviewers. Any product that may be evaluated in this article, or claim that may be made by its manufacturer, is not guaranteed or endorsed by the publisher.
